# Genetic Association of Angiotensin-Converting Enzyme (ACE) Gene I/D Polymorphism with Preterm Birth in Korean Women: Case-Control Study and Meta-Analysis

**DOI:** 10.3390/medicina55060264

**Published:** 2019-06-10

**Authors:** Noo Ri Lee, In Wook Hwang, Hyung Jun Kim, Yun Dan Kang, Jin Wan Park, Han Jun Jin

**Affiliations:** 1 Department of Biological Sciences, College of Natural Science, Dankook University, Cheonan 31116, Korea; gdlove205@naver.com (N.R.L.); hg1427@gmail.com (I.W.H.); laons16@gmail.com (H.J.K.); 2 Department of Obstetrics and Gynecology, Dankook University Hospital, Cheonan 31116, Korea; yundan76@hanmail.net (Y.D.K.); parkdkog@dankook.ac.kr (J.W.P.)

**Keywords:** *ACE*, I/D polymorphism, preterm birth, case-control study, meta-analysis

## Abstract

*Background and Objectives*: The *ACE* gene encodes the angiotensin-converting enzyme (ACE), a component of the renin-angiotensin system. Increased ACE activity may cause abnormal regulation of placental circulation and angiogenesis, resulting in adverse pregnancy outcomes. Previous studies have reported that the insertion/deletion (I/D) polymorphism of the *ACE* gene is associated with the development of preterm birth (PTB). However, results of the association between *ACE* gene I/D and PTB are inconsistent in various populations. Therefore, we performed a case-control study and a meta-analysis to evaluate the association between *ACE* I/D polymorphism and PTB. *Materials and Methods:* We analyzed a total of 254 subjects (111 patients with PTB and 143 women at ≥38 weeks gestation) for the case-control study. For the meta-analysis, we searched Google Scholar, PubMed, and NCBI databases with the terms “ACE,” “angiotensin-converting enzyme,” “preterm birth,” “preterm delivery,” and their combinations. *Results:* Our results of the case-control study indicated that *ACE* I/D polymorphism is significantly associated with PTBs in the overdominant genetic model (odds ratio (OR) 0.57, 95% confidence interval (CI) 0.347–0.949, *p* = 0.029) and that the ID genotype of *ACE* I/D polymorphism has a protective effect for PTB (OR 0.57, 95% CI 0.333–0.986, *p* = 0.043). Similarly, the meta-analysis showed that the OR for the *ACE* gene ID genotype was 0.66 (95% CI 0.490–0.900, *p* < 0.01). *Conclusion:* The *ACE* gene ID genotype has a significant association with PTB and is a protective factor for PTB. A larger sample set and functional studies are required to further elucidate of our findings.

## 1. Introduction

Preterm birth (PTB) is a pregnancy-related disorder defined as a birth before 37 weeks of gestation and is known to increase the risk of infant mortality [1]. According to previous research, approximately 28% of all neonatal deaths are due to PTBs [2]. The incidence rate of PTB is 5–7% of all deliveries and is rising in most developed countries [2]. In the United States, almost 12% of births are preterm [3]. The proportion of PTBs has also risen from 3.8% to 4.7% from 2010 to 2014 in Korea (http://www.koreaherald.com/view.php?ud=20160412000903). However, the pathogenesis of PTB is not well understood [3].

Various factors, such as urogenital infection, abnormal placentation, gestational bleeding, and uterine and cervical anomalies, increase the risk of PTB [4]. Several studies have reported that the pathway for blood pressure regulation is associated with PTB [5,6,7]. In particular, a major function of the renin-angiotensin system is the regulation of uteroplacental blood pressure, and its abnormal function may cause abnormal placentation, leading to gestational disorder [8,9]. To regulate the blood pressure in placenta, angiotensinogen is primarily converted to angiotensin I by renin, and then angiotensin-converting enzyme (ACE) catalyzes the proteolytic conversion of angiotensin I to angiotensin II. Angiotensin II stimulates the secretion of aldosterone, which regulates uteroplacental vasoconstriction and blood pressure [9]. Previous studies have shown that an increase in uteroplacental blood pressure is associated with the development of PTB, preeclampsia, and spontaneous abortion [10,11,12].

The human *ACE* gene is located on chromosome 17q23.3 and has 26 exons and 25 introns [13]. A 287 bp Alu repeat sequence insertion/deletion (I/D) in intron 16 of *ACE* is generally associated with the level of ACE concentration [14]. The DD genotype leads to the highest plasma ACE level, whereas the ID genotype leads to an intermediate level, and the II genotype leads to the lowest level [15]. Increased activity of ACE in placenta was found to be associated with lower uteroplacental blood flow and abnormal placentation that could lead to PTB [16].

A number of studies have analyzed the genetic association between *ACE* gene I/D polymorphism and gestational disorders. Valdez et al. (2007) found that the D allele of *ACE* gene I/D polymorphism was significantly associated with premature rupture of membranes in the Mexican population [17]. Häupl et al. (2015) showed a significant association between *ACE* gene I/D polymorphism and uteroplacental dysfunction in the Middle Eastern Asian population [18]. Uma et al. (2008) observed that the DD genotype of *ACE* gene I/D polymorphism was associated with the development of PTB in the Caucasian population [19]. However, preeclampsia, a disorder that is closely related with PTB, was not associated with *ACE* gene I/D polymorphism in the Turkish population [20]. Similar results were found in the Japanese population [21].

Therefore, we conducted a case-control study and a meta-analysis to investigate the genetic association between preterm birth and *ACE* gene I/D polymorphism. A meta-analysis offers an opportunity to quantitatively review overall association data from individual results of the same genetic polymorphism [22]. A meta-analysis is primarily used to combine inconsistent results of previous studies [22]. This study is the first attempt to analyze the association between *ACE* gene I/D polymorphism and PTB in Korean women and to corroborate the results using a meta-analysis.

## 2. Materials and Methods

### 2.1. Subjects

In this study, a total of 254 women were recruited from the Obstetrics and Gynecology Department at Dankook University Hospital in Korea. One hundred and eleven PTB patients were selected, where the gestational ages were 24–37 weeks [23]. One hundred and forty-three pregnant women with no history of PTB or spontaneous abortion and who were at a gestation of at least 38 weeks were used as the control group. We excluded patients with other systemic diseases, such as hypertension, gestational diabetes, coronary heart disease, placental abruption, chronic nephritis, multiple gestation, and fetal anomalies. All clinical interviews were conducted by an obstetrician, and informed consent was obtained from all the participants of this study. All the participants of this study signed a consent form and were aware of the protocol of this study. The study protocol was approved by the Ethics Committee of the Dankook University Hospital (date of approval and the project identification code are respectively 16 November 2017 and DKUH 2016-12-003-005).

### 2.2. DNA Extraction and Genotyping

DNA was extracted from leukocytes or buccal cells using the GeneAll Exgene Clinic SV mini kit (GeneAll, Seoul, Korea). Genotypes of *ACE* gene I/D polymorphism were detected using PCR. Primer sets were designed according to the previous study reported by Gürdöl et al. [24]: forward 5′-CTGGAGACCACTCCCATCCTTTCT-3′ and reverse 5’-GATGTGGCCATCTTCGTCAGAT-3′ [25]. The PCR reaction was performed in a total volume of 20 µL, comprising 10 ng of genomic DNA, 10 pM of each primer, 0.2 mM dNTPs, 2.0 mM MgCl_2_, 10 × PCR buffer, and 1.0 U NV DNA polymerase (NAVI BioTech, Cheonan, Korea). The PCR amplification was conducted with a C1000 Touch thermal cycler (Bio Rad, Hercules, CA, USA) under the following conditions: 95 °C for 5 min, followed by 40 cycles at 94 °C for 1 min, 64 °C for 1 min, and 72 °C for 1 min, then a final extension at 72 °C for 10 min. Fragments with insertion (I allele) and without insertion (D allele) were detected as 490 and 190 bp, respectively, in 2% agarose gel (Lonza, Morristown, NY, USA).

### 2.3. Search Strategy and Selection Criteria

We performed a meta-analysis of the published literature and present study to analyze the association between *ACE* gene I/D polymorphism and PTB. The PubMed and Google Scholar databases were used as search sources, with the keywords “ACE,” “angiotensin-converting enzyme,” “preterm birth,” “preterm delivery,” “preterm labor,” “premature delivery,” and their combinations. We used the following criteria for selection: English language, human population-based case-control studies, studies for association of *ACE* gene I/D polymorphism and preterm birth, studies consisting of detailed genotype data with proof of Hardy–Weinberg equilibrium (HWE), and studies for only maternal subjects, excluding meta-analysis. Finally, we selected three studies published from 2007 to 2008.

### 2.4. Data Extraction

We extracted the following data from each study: last name of the first author, publication year, subjects’ ethnicity, sample size, number of cases, and control for *ACE* gene I/D genotypes.

### 2.5. Statistical Analysis

An independent *t*-test was performed to compare the following data characteristics: age, height, weight, pre-gestation weight, systolic blood pressure, diastolic blood pressure, birth weight, and gestational age at delivery using SPSS software (ver. 23.0; IBM Corporation, Somers, NY, USA). Chi-squared tests were used to assess HWE. In addition, odds ratios (OR) with 95% confidence intervals (CIs) were calculated in a 2 × 2 table using the genotype and allele frequencies of cases and controls using web-based statistics tools (SISA (http://www.quantitativeskills.com/sisa/) and SNPStats (http://bioinfo.iconcologia.net/SNPStats)). For the meta-analysis, the chi-squared test was performed to prove whether the genotype frequencies in the control group of each reference were assessed for HWE. ORs of each study and pooled data with 95% CIs were calculated to analyze the association between *ACE* gene ID genotype and PTB in the overdominant genetic model with fixed-effect and random-effect models [26]. Because of the small number of included studies, we assumed the heterogeneity by using *I*^2^, i.e., the percentage of the variability [27]. If the *I*^2^ value was over 50%, indicating significant heterogeneity between studies, we selected a random-effect model. If under 50%, we selected a fixed-effect model for analysis to analyze the general effect of the *ACE* gene ID genotype. To investigate evidence for publication bias, we used the test for funnel plot asymmetry described by Egger et al. [28]. In the absence of bias, the funnel plot is shown as a symmetrical shape [29]. All meta-statistical analyses were performed using RevMan ver. 5.3 software (Cochrane Collaboration, Copenhagen, Denmark). A *p* value of <0.05 was considered statistically significant in all statistical analyses.

## 3. Results

### 3.1. Case-Control Study

We analyzed a total of 254 pregnant women. For the mean values of age, height, weight, pre-and post-pregnancy weight, SBP (systolic blood pressure), and DBP (diastolic blood pressure), no meaningful differences were observed between the PTB patients and controls (*p* > 0.05). However, a significant difference was found in birth weight and gestational age between PTB patients and controls (*p* < 0.05) (Table 1).

Genotyping data of the *ACE* gene I/D polymorphism for the 111 patients with PTB and 143 controls are presented in Table 2. The genotype frequencies of the *ACE* gene I/D polymorphism did not deviate from the Hardy–Weinberg equilibrium (Table 2). The ID genotype frequencies were significantly different between PTB women and controls (OR 0.57, 95% CI 0.333–0.986, *p* = 0.043). In the overdominant genetic model, we also found significant association between the ID genotype of *ACE* gene I/D polymorphism and PTB (OR 0.57, 95% CI 0.347–0.949, *p* = 0.029).

### 3.2. Meta-Analysis

**Figure 1:**
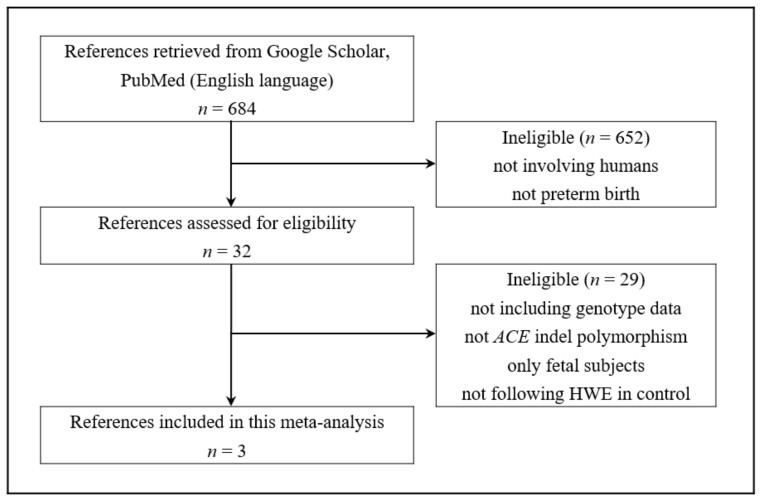
Flow chart of literature identification.

A total of 684 papers were initially searched from the Google Scholar and PubMed databases with the related keywords (Figure 1). Thirty-two studies were identified that potentially analyzed PTB. Among these studies, 29 were ultimately excluded: 18 studies had no genotype data, three did not describe the association with the *ACE* I/D polymorphism, six did not follow the Hardy–Weinberg equilibrium (HWE) in control groups (*p* < 0.05), one used only fetal subjects, and one duplicated the subjects. Finally, we selected four studies with 467 patients and 365 controls, including the present case-control study [17,19,30]. The characteristics of each study included in the meta-analysis are shown in Table 3. 

**Figure 2:**
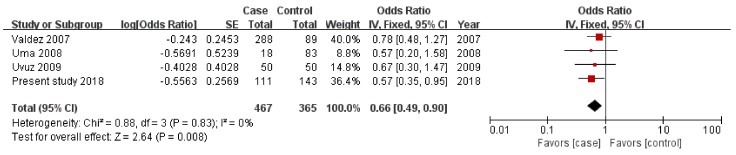
Forest plot of PTB risk associated with ACE I/D polymorphism for a fixed-effect model (over-dominant model; ID genotype vs. II + DD genotype). Squares, study-specific ORs; horizontal line, 95% CI; area of the squares, study specific weight; diamond, pooled results of ORs and 95% CI; ACE, angiotensin-converting enzyme; PTB, preterm birth.

**Figure 3:**
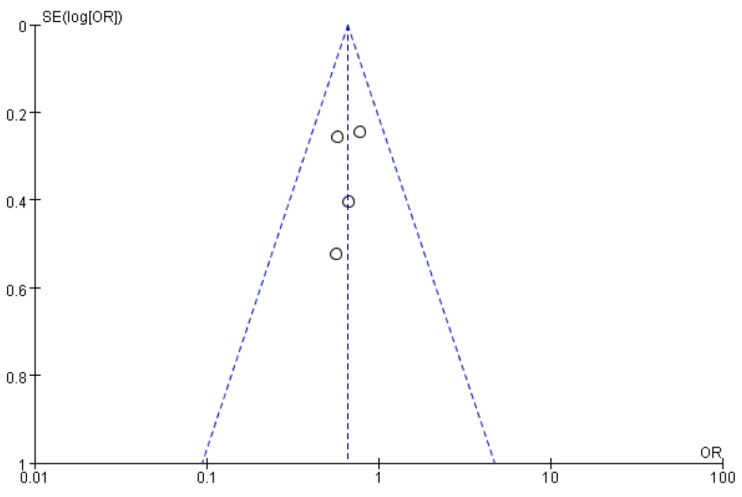
Funnel chart of PTB risk associated with ACE I/D polymorphism.

Our case-control study showed that the ID genotype of *ACE* gene I/D polymorphism is associated with PTB. Therefore, we conducted a meta-analysis for the overdominant genetic model to corroborate this result. The OR for the fixed-effect model was 0.66 (95% CI 0.490–0.9200, *p* = 0.008), indicating that the *ACE* gene ID genotype is protective to PTB and the data indicated no significant heterogeneity between respective studies (*I*^2^ = 0%, *p* = 0.83) (Figure 2). The publication bias is shown schematically with a funnel plot (Figure 3). Each of the scatter points represent the treatment effects from the studies in this meta-analysis [31]. The middle-dotted line shows the average of the estimated ORs in all studies [31]. The diagonal lines on both sides indicate expected 95% confidence intervals around the summary estimate [31]. We confirmed publication bias using Egger’s linear regression test, and the funnel plot did not indicate a distinct asymmetrical result. Therefore, there was no evidence of publication bias in this meta-analysis.

## 4. Discussion

In this study, we evaluated the effect of *ACE* gene I/D polymorphism on PTB. The results of a case-control study showed that the *ACE* gene ID genotype is protectively associated with the development of PTB in Korean women (OR 0.57, 95% CI 0.333–0.986, *p* = 0.043) (Table 2). We also demonstrated an association between *ACE* I/D polymorphism and PTB for the overdominant genetic model (OR 0.57, 95% CI 0.347–0.949, *p* = 0.029) (Table 2).

The results of our case-control study indicated that the D allele and DD genotype are not associated with PTB, but the ID genotype of *ACE* gene I/D polymorphism has a protective effect (Table 2), whereas in previous studies, the D allele had a significantly higher frequency in patients with PTB [5] and the deletion homozygote was a risk factor of abnormal placentation [32]. Fazelnia et al. (2016) suggested that *ACE* gene DD genotype has a significant association with spontaneous recurrent pregnancy loss [33] and Yang et al. (2012) also showed that the D allele of *ACE* gene I/D polymorphism increased the risk of spontaneous recurrent pregnancy loss [34]. Therefore, further analysis is necessary to identify the effect of the D allele and DD genotype on the development of PTB. Several studies have reported that the ID genotype of *ACE* gene has a significant effect on many diseases [35,36,37]. Mata-Balaguer et al. (2004) found that subjects with the ID genotype had intermediate ACE values in the Spanish population, resulting in a protective effect against coronary heart disease [35]. Additionally, Tippisetty et al. (2011) suggested that the ID genotype reduced the risk of the progression of vitiligo compared with the other two genotypes [37]. In addition, Alves Corrêa et al. (2009) suggested that the protective effect of the ID genotype is caused by the co-dominance of alleles [37]. These studies therefore support our finding that the ID genotype of *ACE* I/D polymorphism has a protective effect toward certain diseases. However, the effects of the ID genotype of *ACE* gene I/D polymorphism for PTB have not yet been fully identified. Thus, a functional study into the roles of *ACE* gene I/D polymorphism on PTB is necessary in further studies.

To provide a more precise estimation of the association between *ACE* gene I/D polymorphism and PTB, we conducted a meta-analysis using three studies in addition to present study. As a result, we identified that the ID genotype of *ACE* gene I/D polymorphism has a protective effect on PTB in overall populations as the result of the present case-control study (OR 0.66, 95% CI 0.490–0.900, *p* < 0.008) (Figure 2).

The present study should be interpreted with the following limitations: first, the sample size in this case-control study was not large enough to represent the whole of the Korean population and detect reliably significant associations [38]. A larger sample size is necessary in further replication studies. Second, the number of studies included in this meta-analysis was too small for definite conclusions. Because of the scarcity of data, our results should be verified through further studies. Third, we used only published studies in the meta-analysis. Unpublished and published studies in non-international journals could not be accessed. This factor may cause potential publication bias. Fourth, for accessibility, all the studies in the present meta-analysis were written only in English. This may have affected ethnological heterogeneity. Additionally, it is suggested that both maternal and fetal genetic polymorphism in *ACE* gene with PTB should be determined, but we analyzed only the genotype data from mothers. Finally, PTB is a complex disease caused by the interaction between many factors [37],however, we did not consider gene–gene or gene–environment interactions, such as smoking, alcohol status, social position, and therapy level, which may have an effect on the association between *ACE* gene I/D polymorphism and PTB.

Despite these limitations, our study has many advantages. The association of *ACE* gene I/D polymorphism and PTB has not been extensively researched [19,21], so our case-control study and meta-analysis may corroborate evidence in further studies. In particular, a meta-analysis provides the opportunity to synthesize the similarities and differences among the results of inconsistent studies.

## 5. Conclusions

Our results suggest that the ID genotype of *ACE* gene I/D polymorphism has a significantly protective effect on the pathogenesis of PTB. Similarly, the subgroup analysis also indicated that the polymorphism was related to a decrease in the incidence of PTBs. However, as PTB is multifactorial, our findings require verification with larger sample sizes and functional studies.

## Figures and Tables

**Table 1:** Clinical Characteristics of preterm birth and control groups.

Characteristics	Preterm Birth (*n* = 111)	Controls (*n* = 143)	*p*-Value ^a^
Age (years) (mean ± SE)	30.59 ± 4.59	30.41 ± 4.90	0.779
Height (cm) (mean ± SE)	160.29 ± 4.59	160.56 ± 5.56	0.697
Pre-pregnant weight (g) (mean ± SE)	56.82 ± 9.92	56.10 ± 10.41	0.599
Post-pregnant weight (g) (mean ± SE)	67.37 ± 10.51	69.46 ± 10.45	0.14
SBP (mmHg) (mean ± SE)	120.24 ± 14.97	121.32 ± 14.47	0.585
DBP (mmHg) (mean ± SE)	74.76 ± 12.82	75.79 ± 11.87	0.535
Birth weight (g) (mean ± SE)	2354.51 ± 643.04	3158.46 ± 463.68	<0.001
Gestational age at delivery (weeks) (mean ± SE)	33.19 ± 3.06	38.74 ± 1.06	<0.001

^a^ The *p*-value is for *t*-test.

**Table 2:** Genotype and allele frequencies of *ACE* I/D polymorphism in the preterm-birth patients and control group.

Marker			Preterm-Birth (*n* = 111)	Control (*n* = 143)	*p*-Value ^b^	OR (95% CI)
*ACE* I/D	Genotype	II	50 (45.1)	50 (35.0)		Reference
		ID	43 (38.7)	75 (52.4)	0.043 *	0.57 (0.333–0.986)
		DD	18 (16.2)	18 (12.6)	0.999	1.00 (0.467–2.142)
	Allele	I	143 (64.4)	175 (61.2)	0.456	
		D	79 (35.6)	111 (38.8)		
	*p*-value ^a^		0.102	0.212		
	Dominant	II	50 (45.0)	50 (35.0)	0.100	0.66 (0.395–1.090)
		ID + DD	61 (55.0)	93 (65.0)		
	Recessive	II + ID	93 (83.8)	125 (87.4)	0.410	1.34 (0.663–2.724)
		DD	18 (16.2)	18 (12.6)		
	Overdominant	II + DD	68 (61.3)	68 (47.5)	0.029 *	0.57 (0.347–0.949)
		ID	43 (38.7)	75 (52.5)		

^a^ The *p*-value is for Hardy–Weinberg equilibrium (HWE). ^b^ The *p*-value is for chi-square test. * *p* < 0.05.

**Table 3:** Characteristics of the studies included in the meta-analysis.

Authors	Year	Country	Ethnicity	No. of Cases	No. of Controls	Case (Genotype)		Control (Genotype)		HWE in Control (*p*)	Genotyping Method
						ID	II + DD	ID	II + DD		
Valdez et al.	2007	Mexico	American	288	89	151	137	52	37	0.1115	Sequencing
Uma et al.	2008	UK	Caucasian	18	83	9	9	53	30	0,1557	PCR
Uvuz et al.	2009	Turkey	Caucasian	50	50	21	29	26	24	0.3636	PCR
Present study	2018	Korea	Asian	111	143	43	68	75	68	0.2125	PCR
